# Modular reorganization of brain resting state networks and its independent validation in Alzheimer's disease patients

**DOI:** 10.3389/fnhum.2013.00456

**Published:** 2013-08-09

**Authors:** Guangyu Chen, Hong-Ying Zhang, Chunming Xie, Gang Chen, Zhi-Jun Zhang, Gao-Jun Teng, Shi-Jiang Li

**Affiliations:** ^1^Department of Biophysics, Medical College of WisconsinMilwaukee, WI, USA; ^2^Department of Radiology, Jiangsu Key Laboratory of Molecule Imaging and Functional Imaging, Medical School of Southeast UniversityNanjing, PR China; ^3^Department of Radiology, Subei People's Hospital of Jiangsu Province, Yangzhou UniversityYangzhou, PR China; ^4^Department of Neuropsychiatry, Affiliated Zhong Da Hospital of Southeast UniversityNanjing, PR China; ^5^Department of Psychiatry and Behavioral Medicine, Medical College of WisconsinMilwaukee, WI, USA

**Keywords:** Alzheimer's disease, MCI, validation, module analysis, resting-state functional connectivity, brain network, gray matter concentration, graph theory

## Abstract

Previous studies have demonstrated disruption in structural and functional connectivity occurring in the Alzheimer's Disease (AD). However, it is not known how these disruptions alter brain network reorganization. With the modular analysis method of graph theory, and datasets acquired by the resting-state functional connectivity MRI (R-fMRI) method, we investigated and compared the brain organization patterns between the AD group and the cognitively normal control (CN) group. Our main finding is that the largest homotopic module (defined as the insula module) in the CN group was broken down to the pieces in the AD group. Specifically, it was discovered that the eight pairs of the bilateral regions (the opercular part of inferior frontal gyrus, area triangularis, insula, putamen, globus pallidus, transverse temporal gyri, superior temporal gyrus, and superior temporal pole) of the insula module had lost symmetric functional connection properties, and the corresponding gray matter concentration (GMC) was significant lower in AD group. We further quantified the functional connectivity changes with an index (index A) and structural changes with the GMC index in the insula module to demonstrate their great potential as AD biomarkers. We further validated these results with six additional independent datasets (271 subjects in six groups). Our results demonstrated specific underlying structural and functional reorganization from young to old, and for diseased subjects. Further, it is suggested that by combining the structural GMC analysis and functional modular analysis in the insula module, a new biomarker can be developed at the single-subject level.

## Introduction

Alzheimer's disease (AD) is considered a disconnection syndrome (Geschwind, 1965; Delbeuck et al., 2003). Recent studies demonstrated that the underlying neural mechanisms responsible for the disconnection syndrome are involved in the functional disruption in the brain of AD patients (Horwitz et al., 1987; Wada et al., 1998). An increasing number of studies have focused on imaging the default mode network (DMN) in aging and dementia by using intrinsic blood oxygenation level-dependent (iBOLD) signals, acquired by the resting-state functional MRI (R-fMRI) method (Lustig et al., 2003; Greicius et al., 2004; Sorg et al., 2009; Khalili-Mahani et al., 2012). The measurement of functional disruption in the DMN could become a potential clinical diagnostic biomarker for AD because convergent evidence demonstrated that brain atrophy, Aβ-amyloid plaque deposition and metabolic deficits co-occurred in the DMN (Buckner et al., 2009). Several other studies demonstrated that functional disruption also occurred in other areas besides the DMN, such as the hippocampus and the insular networks (Li et al., 2002; Bonthius et al., 2005; Royall, 2008; Xie et al., 2012). However, despite these scientific advancements, efforts to cross-validate the functional disruption trait as a biomarker have been of limited success.

Specifically, several studies provided the diagnostic power of the DMN for AD (Li et al., 2002; Greicius et al., 2004; Fleisher et al., 2009; Koch et al., 2010, 2012), but follow-up studies by other research groups are either lacking (Li et al., 2002; Greicius et al., 2004; Fleisher et al., 2009; Koch et al., 2010, 2012), controversial (Zhang et al., 2009; Yu et al., 2011), or failed to confirm a solid diagnostic value (Prvulovic et al., 2011). As a result, despite the efforts during the past decade, there is no robust biomarker based on R-fMRI technology, which has substantially limited its potential utility value in AD research and treatment. There are several factors that may contribute to the current stagnant status. First, in typical seed-based R-fMRI studies, the group-level *t*-tests often statistically identified the connectivity maps that highlight voxels where functional connectivity is disrupted. Such a statistical approach often overestimates the diagnostic power, even if the leave-one-out approach or seven-fold cross-validation method is employed (Chen et al., 2011a; Westman et al., 2012a,b). Second, because of compensatory mechanisms or increased activation, brain connectivity may be reorganized along the continuum of disease progression (He et al., 2008; Sanz-Arigita et al., 2010). Not only did the functional connectivity decrease in certain regions, but it also increased in other regions (Zhang et al., 2010). As a result, the summation of the overall connectivity strength may not change significantly. Third, the disconnection syndrome in AD may be the result of the functional and structural disruptions in the large-scale networks; therefore, the seed-based network alone, such as the DMN, may have no sufficient power as a biomarker. As a result, when applying trained classifiers to independent datasets, the specificity and sensitivity were low.

To overcome these shortcomings and to move the research field forward, the present study is focusing on three new approaches. First, we extend the seed-based analysis to the modular analysis method (He et al., 2009; Meunier et al., 2009a,b, 2010) to examine the patterns of brain network reorganization at the large-scale network level to test the hypothesis that the AD network organization is a reconfiguration from CN networks where some subnetworks that are related to cognitive processing may change and others are preserved. A previous study (Faria et al., 2012) addressed the factor that network (or called Atlas)-based analysis can enhance SNR and reproducibility of resting-state functional connectivity. In addition, in using network-based functional connectivity, the number of false positive cross-correlations can be significantly reduced due to the reduced number of the total pairs of correlations. To our knowledge, the applicability of the modular analysis to examine the resting-state functional network reorganization pattern in mild cognitive impairment (MCI) and AD brains has not been demonstrated. Second, based on specific changes in brain reorganization patterns at the module level, an exploratory analysis was performed to evaluate if the changes can be employed as a biomarker for AD. Third, we employed an additional six independent R-fMRI datasets from human subjects to independently cross-validate the module-based biomarker at the single-subject level.

## Materials and methods

### Human subjects

A total of 331 subjects in eight groups were employed for this study. Two R-fMRI datasets obtained from the cognitively normal (CN) group (*N* = 30) and the mild AD group (*N* = 30) from the Medical College of Wisconsin (MCW) site (referred to herein as MCW datasets) (Table 1) were employed as the testing datasets to identify changes in the modular reorganization patterns occurring in AD brains as a biomarker. We then employed six additional independent R-fMRI datasets to cross-validate the biomarker. Among the six sets of datasets, one was obtained from amnestic mild cognitive impairment (aMCI) subjects (*N* = 23) from the MCW site, three datasets were obtained from a group of 56 elderly subjects from Southeast University, Nanjing, China, comprised of elderly CN subjects (*N* = 20), aMCI subjects (*N* = 22), and AD subjects (*N* = 14) (referred to herein as Nanjing datasets) (Table 1) (Zhang et al., 2010). The other two independent R-fMRI datasets are comprised of 192 young subjects; these were downloaded from the 1000 Functional Connectomes Project database (www.nitrc.org/projects/fcon1000/) from Beijing Zang's datasets (http://www.nitrc.org/frs/shownotes.php?release_id=819) (referred to herein as Beijing datasets) (Table 1) (Biswal et al., 2010). All of these subjects were obtained from databanks. For detailed subject information, please refers to originally published papers (Biswal et al., 2010; Zhang et al., 2010; Chen et al., 2011a).

**Table 1:** **Summary of demographic information for test and validation groups of subjects**.

**Groups**	**Number**	**Age**	**Male/Female**	**MMSE**	**Education (year)**	
CN_3T	30	75.9 ± 6.42	16/14	29.4 ± 1.03	NA	Test group
AD_3T	30	76.7 ± 5.28	17/13	24.8 ± 2.97	NA	
MCI_3T	23	76.1 ± 6.84	11/12	27.83 ± 1.67	NA	Validation group
CN_1.5T	20	68.9 ± 6.44	7/13	28.6 ± 1.05	10 ± 3.71	Validation group
MCI_1.5T	22	71.6 ± 4.95	10/12	27.2 ± 1.4	10.7 ± 3.50	Validation group
AD_1.5T	14	71.3 ± 5.09	5/9	22.2 ± 2.91	9.6 ± 4.96	Validation group
Young 18–22 3T	150	20.4 ± 1.13	59/91	NA	NA	Validation group
Young 23–26 3T	42	23.9 ± 1.02	27/15	NA	NA	Validation group

### Imaging acquisition of MCW datasets

Imaging was performed using a whole-body 3T Signa GE scanner with a standard quadrature transmit receive head coil. During the resting-state acquisitions, no specific cognitive tasks were performed, and the study participants were instructed to close their eyes and relax inside the scanner. Sagittal resting-state functional MRI (fMRI) datasets of the whole brain were obtained in 6 minutes with a single-shot gradient echo-planar imaging (EPI) pulse sequence. The fMRI imaging parameters were: TE of 25 ms, TR of 2 s, flip angle of 90°; 36 slices were obtained without gap; slice thickness was 4 mm with a matrix size of 64 × 64 and field of view of 24 × 24 cm. High-resolution SPGR 3D axial images were acquired for anatomical reference. The parameters were: TE/TR/TI of 4/10/450 ms, flip angle of 12°, number of slices of 144, slice thickness of 1 mm, matrix size of 256 × 192. To make sure that cardiac and respiratory frequencies did not account for any significant artifacts in the low-frequency spectrum, a pulse oximeter and respiratory belt were employed to measure these physiological noise sources. Further processing ensured a minimizing of the potential aliasing effects.

### Imaging acquisition of beijing datasets

The data was acquired at 3T Siemens Scanner. We used 192 subjects out of a total of 198 young subjects from Beijing Zang's datasets. Six subjects were discarded during the preprocessing procedures for a variety reasons. The imaging acquisition parameters can be found on the website (http://www.nitrc.org).

### Imaging acquisition of nanjing datasets

The data was acquired at 1.5T Philips Scanner. Subjects wore headphones and were instructed to lie in a supine position in a standard head coil of a 1.5-T MR imaging unit (Eclipse; Philips, Best, The Netherlands). Structural images were obtained. Resting-state functional images were acquired by using a gradient-echo EPI sequence (TR/TE, 3000/40 ms; flip angle, 90°, slice thickness, 6 mm; slice gap, 0 mm; field of view, 240 mm; and matrix size, 64 × 64; 18 axial slices and 128 time points). For detailed parameters and demographic information, please refer to previous study (Zhang et al., 2010). All of these studies were conducted with Institutional Review Board approval and were in compliance with Health Insurance Portability and Accountability Act (HIPAA) regulations or similar polices in China.

### Data preprocessing

We used Analysis of Functional NeuroImages (AFNI) software (http://afni.nimh.nih.gov/afni/) and MATLAB (Mathworks) in this study for data processing. The first five volumes of each raw resting-state functional imaging dataset were discarded to allow for T1 equilibration. Interleaved slice acquisition-dependent time shifts were corrected (AFNI command, *to3d -time:zt nz nt TR tpattern*). Spikes in time series data were removed (AFNI command, *3dDespike*). Data were then motion corrected (Six motion parameters, including roll, pitch, in the superior, left and posterior direction displacement were estimated by volume registration of the R-fMRI data, and then, were regressed out by using Afni command 3dDeconvolve to control possible micromovement effects). There was no group difference for movement parameters. Detrend processing procedure using AFNI commands (*3dvolreg and 3dDetrend*) was performed. The reference template in Talairach space, which contained 116 anatomically defined regions of interest (ROIs) (Tzourio-Mazoyer et al., 2002), was transformed and aligned to the SPGR images and EPI resting-state functional images for each subject (AFNI command, *3dfractionize*). This resulted in 116 mapped ROIs. The average time course within each ROI was extracted from the resting-state functional imaging datasets. Averaged white matter signal and cerebrospinal fluid (CSF) signal were extracted using white matter mask (http://afni.nimh.nih.gov/pub/dist/data/TT_wm+tlrc) and CSF (http://afni.nimh.nih.gov/pub/dist/data/TT_csf+tlrc) mask in Talairach space. These two masks were transformed and aligned to the SPGR and echo planar images for each subject (AFNI command, *3dfractionize*). Then, the average time courses within the CSF or the eroded white matter mask, together with global mean signals, were removed as nuisance regressors from the 116 regional time courses with linear regression using Matlab (Mathworks).

### Postprocessing

#### Brain functional network

We constructed a region-wise whole-brain resting-state functional network for each subject. A network size is *N* (*N* is the num*ber of Nodes or ROIs, in this study, N = 116)*, there are *N × (N – 1)/2* possible edges in a fully connected network expressed in a matrix. The weighted strength of each edge between nodes *i* and *j* was defined as CC_ij_ (cross-correlation coefficient (**CC)** between two time series of *ROI(i)* and *ROI(j)*). The weighted distance of each edge between a pair of directly connected nodes *ROI(i)* and *ROI(j)* was defined as *d*_*ij*_ = 1 − *CC*_*ij*_. The adjacent matrix of *CC*_*ij*_ represents graph *G*, such that *G* = {*V, S, D*}, consisting of a set of vertices(Nodes) *V* = {*V*_1_, *V*_2_, …, *V*_*N*_}, a set of edges *S* = {*CC*_*ij*_|*i, j* = 1, 2, …, *N*} and a set of associating weighted edge distances D = {1 − CC_i, j|i, j = 1, 2, …,N} between brain regions *ROI(i)* and *ROI(j)*.

#### Group network

A group functional network matrix **(A)** is constructed by the ratio of mean to the standard deviation of all individuals' matrices in this group. Each element value of **A** is calculated as follows:

(1) $$a_{ij}=\frac{\frac{1}{n}\sum_{k=1}^{n}CC_{k,i,j}}{\sqrt{\frac{1}{n}\sum_{k=1}^{n}{\left(CC_{k,i,j}-\mu_{ij}\right)}^{2}}}$$

This matrix can reduce the intersubject variation of the functional connectivity especially those connections with large intersubject variation. *k* is the subject number, *n* is the number of subjects, *i* and *j* are two ROIs of *ROI(i)* and *ROI(j)*. In this study, we only use the positive CC value in group network as previously described (Chen et al., 2011b) for further modular analysis.

#### Modularity

Module is defined as a community, the inside of which has denser connections than the rest of the network (Newman and Girvan, 2004). Several algorithms have been developed to detect those modules (Clauset et al., 2004; Duch and Arenas, 2005). The basic approach is to measure the maximum modularity value, Q, which is defined as:

(2) $$Q=\frac{1}{2m}\sum_{i,\;j}\left[a_{ij}-\frac{k_{i}k_{j}}{2m}\right]\delta (c_{i},c_{j}).$$

*a*_*ij*_ is the adjacent weighted matrix which represents the network, *m* is the number of connections in the network, and *k*_*i*_ is the degree of node *i* (Ahnert et al., 2007) and *c*_*i*_ is the module *i*.

In order to find the communities in the brain functional network, we use the spectral algorithm of Newman (Newman, 2006; Leicht and Newman, 2008), which is implemented in the Brain Connectivity Tool Box (https://sites.google.com/a/brain-connectivity-toolbox.net/bct/Home). This program can find the network organization pattern with the best modularity value (Q).

#### Quantitative measurement of modular reorganization in AD

Based on our hypothesis that AD may reorganize modular patterns compared to CN, the reorganization pattern may exhibit the disruption properties of the whole-brain function network. In order to quantify the changes in the modular patterns in the subnetworks, we created two functional indices (index A and index B) to measure the inter- and intra-hemisphere connections. Index A measures an average of functional connectivity strength in homotopic pairs (*ROI_L*(*i*), *ROI_R*(*i*))

(3) $$\text{indexA}=\frac{1}{n}\sum_{i=1}^{n}CC\left(ROI\_L(i),ROI\_R(i)\right)$$

Where *ROI_L*(*i*) and *ROI_R*(*i*) are two corresponding bilateral homotopic regions, and *CC*(*ROI_L*(*i*), *ROI_R*(*i*)) is the two time courses CC value of *ROI_L*(*i*) and *ROI_R*(*i*). The *n* is the number of pairs of homotopic regions to calculate index A. Index B is used to measure an average of functional connectivity strength within selected unilateral ROIs.

(4) $$\begin{array}{l} \text{indexB}=\frac{1}{n}(\sum_{i=1}^{n}\overset{n}{\underset{j=1}{\sum }}CC\left(ROI\_L(i),ROI\_L(j)\right)\\ \;+\sum_{i=1}^{n}\overset{n}{\underset{j=1}{\sum }}CC\left(ROI\_R(i),ROI\_R(j)\right)) \end{array}$$

There are n ROIs from the right hemisphere (ROI_R) and n corresponding homotopic ROIs from the left hemisphere (ROI_L).

#### Gray matter concentration (GMC)

Besides the functional connectivity, we also calculated the GMC on the AAL template within the regions that showed functional disruption. The gray matter of each subject is segmented by using SPM 8 software (www.fil.ion.ucl.ac.uk/spm/software/spm8/) and then normalized into Talairach space to extract each part of the regions using AFNI and 116 AAL templates. GMC value of each region is the average overall voxel values within those regions involved in modular reorganization.

## Results

### Maximum modularity value (Q)

**Figure 1:**
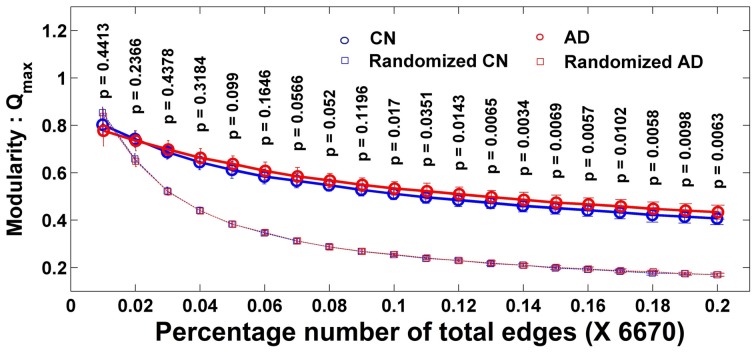
**Individual modularity values (Q) distribution of a network as the function of the number of edges and its corresponding random network**. Error bar shows the standard deviation. All groups' networks have a significantly larger Q-value than the corresponding random networks. Larger Q shows the ability of a network to form modules.

The Q is determined with the modular algorithm, which measures how a network can be separated into different subnetworks. With the MCW dataset, Figure 1 shows the averaged maximum modularity values of individual subjects in each group as a function of number of edges (NE). All subjects in groups CN and AD have larger averaged Q values than their corresponding random networks, indicating that their complex functional networks have a strong ability to form modules, and the module analysis method can be applied to disease populations, such as MCI and AD. There is no group difference in Q value at all different thresholds of NE after the familywise error correction.

### Module structures in the AD group were reorganized, unlike the CN group

**Figure 2:**
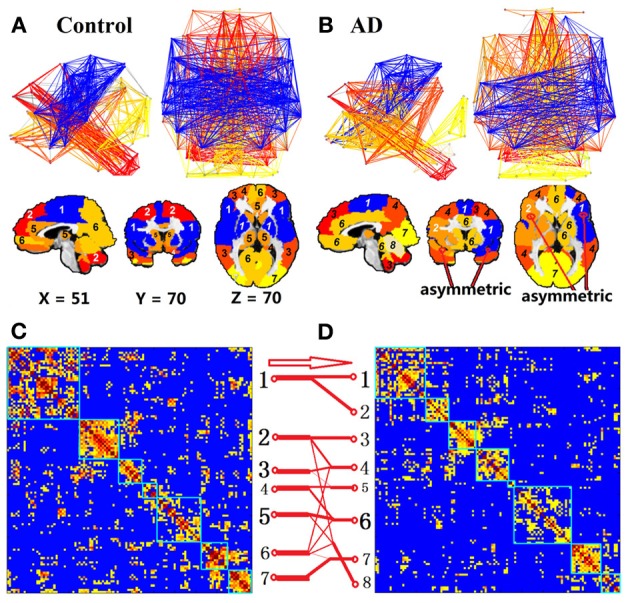
**Module of the non-threshold positive group networks of CN and AD at 3T**. Color represents the individual community. First row of **(A)** and **(B)** is the network view, the second row of **(A)** and **(B)** shows the brain module organization overlaid in the brain template and the last row (**C** and **D**) is the module reorganization pattern between CN and AD. The label module numbers in the brain views of **(A)** and **(B)** are matched with the module numbers in **(C)** and **(D)**, respectively. Two matrices, **(C)** and **(D)**, show the grouped CC matrix of CN and AD. In **(C)** and **(D)**, numbers along each matrix labeled the module number for each group. Red arrow and red connection lines show the reorganization pattern from CN to AD. The thickness of each line represents the number of members.

Although the complex functional network of the AD group has the ability to form the modular structures, similar to the CN group, the modular patterns and membership are quite different between the CN and AD groups, and demonstrated network reorganization patterns. The modular structures are expressed into two forms of presentations: the graphic presentation and mapping presentation, as illustrated in Figures 2A,B for CN and AD groups, respectively. For the CN group, the brains were organized into seven modules. For the AD group, the brain modules were reorganized into eight modules. The module-reorganization patterns between CN and AD are graphically illustrated in Figures 2C,D. The largest module in the CN group (CN-1) was broken down into two separated modules in the AD group (AD-1 and AD-2). The module CN-2 is disrupted into three modules (AD-3, AD-4, and AD-8) and module CN-6 is disrupted into four modules (AD-4, AD-6, AD-7, AD-8).

To compare the module membership composition between CN and AD groups and identify specific brain regions that disrupted away from the original module, the module CN-1 was cited as an example. As listed in Table 2, the module CN-1 contained eight pairs of homotopic brain regions, which are defined as geometrically symmetric across interhemispheric regions. This well-organized module is highly symmetric across hemispheres in the control network (Figure 2A). We called this module the “insula module,” because its members are involved in saliency, switching, attention and control functions of the insula network (Menon and Uddin, 2010). Noticeably, eight out of 16 homotopic regions were broken in the AD group. The eight regions on the right hemisphere formed a new module in the AD group (AD-2) (labeled in Red bold in Table 2). These eight regions are the right opercular part of inferior frontal gyrus, right area triangularis, right insula, right putamen, right globus pallidus, right transverse temporal gyri, right superior temporal gyrus, and right superior temporal pole. The formation of the new module AD-2 not only indicated that the insula module is broken down, but also indicated there is severe disruption between left and right hemisphere communication in the AD brains.

**Table 2:**
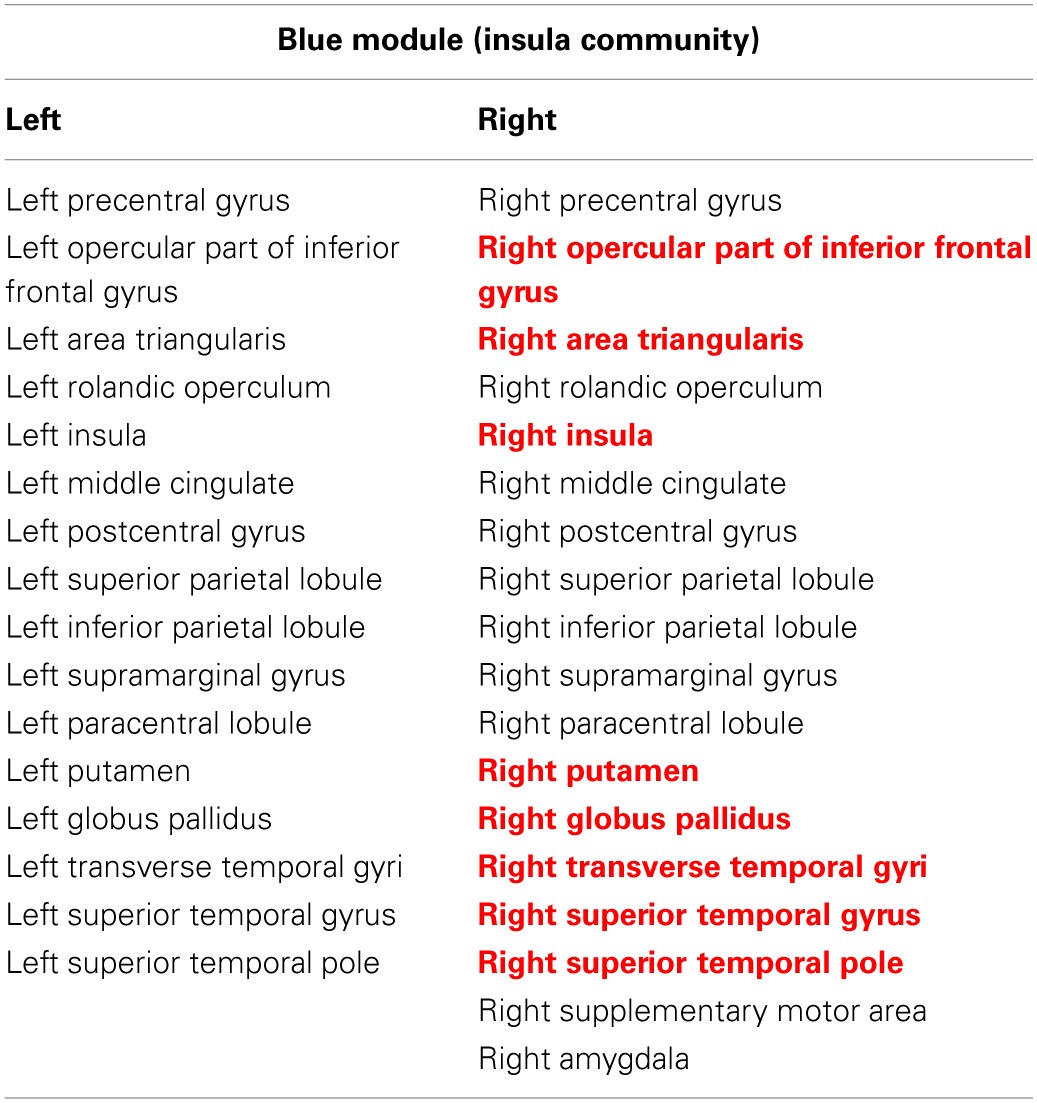
**Regions in the insula module (blue module)**.

The eight right brain regions (in red bold) are no longer the members of the blue module in the AD brains.

### Quantification and validation of the insula module in healthy young, CN, MCI, and AD groups

**Figure 3:**
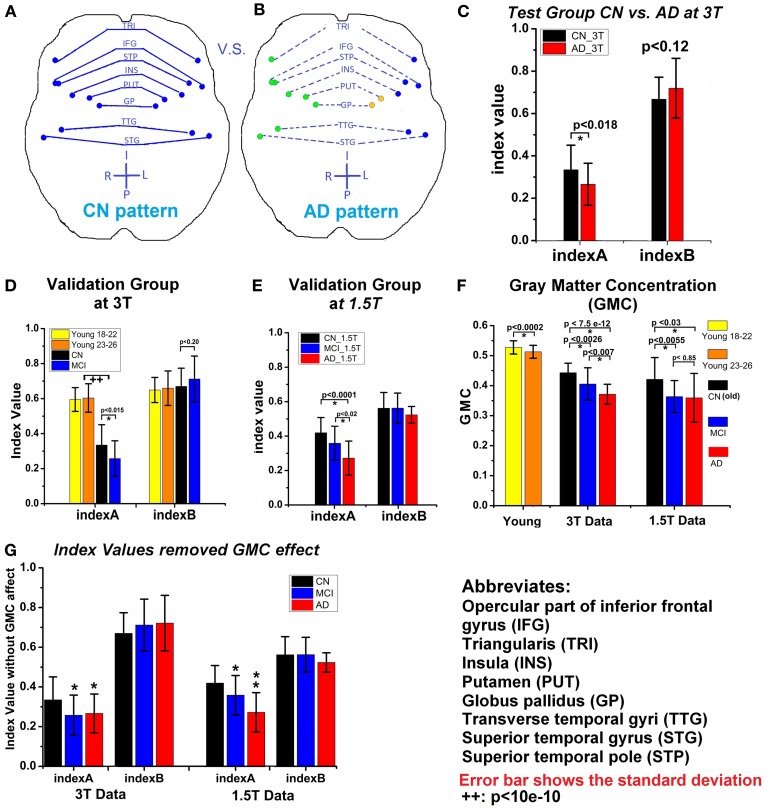
**(A)** and **(B)** Show the changes of insula module between CN **(A)** and AD **(B)**; each dot is the center of an AAL-based brain region; regions with same color are in the same module, solid line means higher connection, while the dash line represents the weaker connection. **(C)** Two indices (index A and index B) of CN_3T and AD_3T (test groups). **(D)** Two indices of two young subject groups, CN_3T and MCI_3T (validation groups). **(E)** Two indices of CN_1.5T, MCI_1.5T, and AD_1.5T (validation groups). **(F)** The average of GMC of each group. **(G)** The two indices after removal of the GMC effect of each group; where (^*^) represents the significance (*p* < 0.05) in comparison to CN_1.5T and (^**^) represents the significance (*p* < 0.05) in comparison to the MCI group. Error bar indicates the standard deviation. (^++^) represents both young groups have significant larger index A value than the old CN_3T and MCI_3T groups.

To quantify the insula module disruption between hemispheres, two functional indices were obtained [index A calculated from Equation (3) and index B calculated from Equation (4)]. As illustrated in Figures 3A,B, functional connections of the eight pairs of homotopic (contralateral) regions of the insula module were disconnected in the AD group. As shown in Figure 3C, index A is significantly (*p* < 0.018) decreased in the AD group compared to the CN group. Index B shows no significant difference related to the AD and CN groups but has increasing trends (*p* < 0.12). To cross-validate the results with indices A and B, we employed six additional independent datasets in order to avoid an *overly optimistic* estimate of the error rate by the *resubstitution method* or the *leave-one-out method*. First, we employed the MCW dataset containing 23 aMCI subjects. As shown in Figure 3D, index A, individually calculated from each aMCI subject in the aMCI group, was significantly lower than that in the CN group and index B showed no difference. Second, we employed the Beijing datasets containing the young groups of subjects (group age between 18 and 22 years old, and group age between 23 and 26 years old, total 192 subjects). As shown in Figure 3D, index A of young subjects has stronger homotopic connectivity strength than that of the elderly CN subjects and no differences for index B. Third, we further demonstrated that datasets acquired on the 1.5T scanner can be employed to validate our results. With the Nanjing datasets acquired from 1.5T scanner, index A of the MCI and AD groups is significantly reduced compared to the CN group, as shown in Figure 3E. These validated results demonstrated that index A as a biomarker can be quantitatively employed for monitoring AD progression in the continuum of disease processes: higher index A in young, decrease in elderly CN groups, and more significantly decreased in the MCI and AD groups.

Decreased gray matter concentration (GMC) of these eight pairs of homotopic regions in MCI and AD groups. The disrupted functional connectivity occurred in the eight pairs of homotopic regions in the insula module. In addition, the average gray matter concentration (GMC) of those regions showed significant decrease in the MCI and AD groups in comparison to the CN groups. The GMC decrease in the MCI and AD group was observed, as shown in Figure 3F. To determine if the GMC changes affect the calculation of index A, the variance of the GMC factor was controlled out. As shown in Figure 3G, index A is still valid in distinguishing between CN from MCI or AD status. These results also indicated that although structural density and functional connectivity decrease may be related, their changes are not necessary proportional (Palop et al., 2006). With this trait, we have combined index A and GMC to examine their diagnostic potential, as described below.

**Figure 4:**
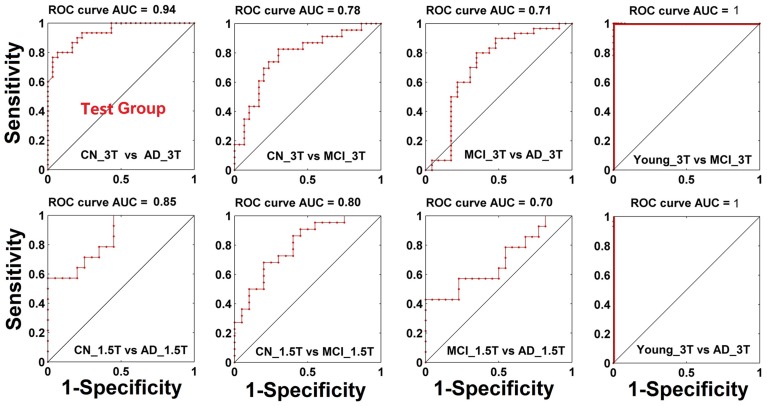
**Classification power (ROC curve) when both biomarkers of GMC and index A are combined**.

Diagnostic power of index A and GMC as biomarkers to classify CN, MCI, and AD statuses. Through the measurement of index A and GMC on each single subject, we have explored their potential as biomarkers to classify CN, MCI, and AD statuses. As shown in Figure 4, the result from testing groups (CN vs. AD) provided 94% of area under the curve (AUC) of the receiver operation characteristic (ROC) curve. The validation results provided 78 and 71% of AUC to classify between CN and AD, and between MCI and AD, respectively. With Nanjing datasets acquired on the 1.5T Siemens scanner, these validation results become 85% (AD vs. CN) and 80% (MCI vs. CN), and 70% (MCI vs. AD). For the young subject groups as the healthiest population, there is a perfect 100% specificity and sensitivity (AUC 100%) in comparison to the AD group.

## Discussion

Several studies have employed the modular analysis method to demonstrate that the brain has modular organization (Hilgetag et al., 2000; Chen et al., 2008; Hagmann et al., 2008). In comparison to small-world metrics, modular analysis can provide detailed network organization patterns as to how the nodes are connected to form subnetworks or communities in a complex network (Hilgetag et al., 2000; Chen et al., 2008; Hagmann et al., 2008). Using this advantage, modular analysis methods have been applied to diseased resting-state brain networks, such as in chronic back pain (Balenzuela et al., 2010) and schizophrenia (Alexander-Bloch et al., 2010; Yu et al., 2011). Using magnetoencephalography (MEG), it was also found that the module strength and the number of modules significantly changed in AD patients (de Haan et al., 2012). Our results are consistent with these findings and demonstrated the applicability of R-fMRI datasets for modular analysis to AD.

In the control network, as expected, module patterns are well organized with symmetric distribution. Each pair of the interhemispheric homotopic regions, for the most part, is in the same communities. Many literature references substantiate that the brain functional network forms an interhemispheric symmetric pattern with highly consistent functional connectivity between homotopic regions (Zuo et al., 2010). A high degree of symmetry in the motor cortex of resting-state functional connectivity has been reported (Biswal et al., 1995; Van den Heuvel and Hulshoff Pol, 2010). The well-known DMN (Raichle et al., 2001; Greicius et al., 2004) has a symmetric, well-organized pattern. Similar to the module method, Mezer (Mezer et al., 2009) used the clustering method and discovered a symmetric pattern of clusters between the two hemispheres. This was true not only in the human brain, but also in the rat brain. The highest values of functional connectivity exist between interhemispheric homotopic regions (Pawela et al., 2008, 2010). However, not all the interhemispheric homotopic regions are symmetric, only some regions and their homotopic regions belong to different communities. This may be due to the dynamic changes of the functional connectivity (Chang and Glover, 2010).

As expected in mild AD group, some of the communities lost symmetric properties. There are more single regions whose homotopic regions are in different communities. For example, module 1 (blue module in Figure 2A) in the control group is very symmetric, while it is separated into two modules in the AD group (blue and brown modules in Figure 2B). Decreased symmetric properties or functional connectivity between interhemispheric homotopic regions have been found in many diseased functional networks. In behavioral research, Yamina (Lakmache et al., 1998) found that AD subjects performed normally when using intrahemispheric processing, but did poorly when interhemispheric communication was required. For instance, in imaging research, EEG studies (Locatelli et al., 1998; Babiloni et al., 2004) of AD found decreased coordination between interhemispheric networks. In the cocaine-dependent group, Kelly (Kelly et al., 2011) investigated the interhemispheric homotopic connections using the Voxel-Mirrored Homotopic Connectivity method, and found the striking cocaine-dependence-related reduction in interhemispheric resting-state functional connectivity among nodes of the dorsal attention network. Also, decreased interhemispheric functional connectivity in subjects with impaired awareness were found (Ovadia-Caro et al., 2012). Therefore, this phenomenon of losing symmetric properties may reflect the cognitive decline and unbalanced state in the functional network of the diseased brain.

The most significant finding of this module study is the interrupted integration of insula module in AD group. Anatomically, the insula is a crucial hub in the human brain network; it is widely connected to the cortical, limbic, and paralimbic structures. Functionally, it is involved in high-order cognition, emotion, autonomic, and sensory process (Naqvi et al., 2007; Allen et al., 2008). The previous study has shown that the insula was affected in AD and its atrophy was significantly decreased from the normal population (Fan et al., 2008). The seed-based functional connectivity of the insular regions was discovered to be significantly decreased in the regions that functionally connected with insula. This disruption was associated with episodic-memory deficits in aMCI patients (Xie et al., 2012). Our results are not only consistent with these previous findings, they indicate a disruption between the insula and other brain regions. Also, we detected the breakdown of the insula module in the AD group, which is a possible neural underpinning of AD dementia.

Our findings demonstrated that the specific reorganized modular patterns can be quantified with index A in the CN, MCI, and AD groups. Unlike biomarkers with inverse U-shape patterns, such as the fMRI method due to the compensatory mechanisms (Dickerson and Sperling, 2009), index A is a monofunction with the disease progression of AD. Index A of aMCI and AD subjects is significantly lower than that of CN and young subjects. Because the biggest risk factor of AD is aging, the congruency between the changes in the index A value, and changes in age, demonstrated the potential of index A to serve as a biomarker. This characteristic of the monofunction of index A with age is very important for diagnostic accuracy by decreasing false positive and negative errors.

We showed the potential of using structural changes (GMC) and functional disruption in the insula module (index A) as a biomarker for AD. Recent revision of the NINCDS-ADRDA (National Institute of Neurological and Communicative Disorders and Stroke and the AD and Related Disorders Association [now known as the Alzheimer's Association]) criterion for the diagnosis of AD suggested adding abnormal biomarkers, such as MRI, positron emission tomography (PET), CSF, and brain atrophy to strengthen their roles (Kohannim et al., 2010; Nettiksimmons et al., 2010; Walhovd et al., 2010; McKhann et al., 2011; Zhang et al., 2011; Dai et al., 2012; Ewers et al., 2012; Westman et al., 2012b). The effective combination of these biomarkers can clinically provide more diagnostic power than using a single biomarker. In this study, we found that the combination of MRI atrophy biomarker and the R-fMRI biomarker of insula module could enhance the classification of AD and monitor the progression along the continuum of AD development both in the test and validation group. Our results demonstrated the great feasibility of combining both MRI-based biomarkers of the insula module in AD diagnosis.

In summary, with the modular analysis, we demonstrated the ability of index A and GMC of the insula module in distinguishing MCI and AD from old and young, healthy CN subjects, and its power of cross-validation with six independent datasets. The combination of the MRI-based structural biomarker and functional biomarker will significantly enhance the diagnostic power. Further studies will be needed to characterize the relationships between different biomarkers for AD (Sperling et al., 2009; Kohannim et al., 2010; Nettiksimmons et al., 2010; Sheline et al., 2010a,b; Walhovd et al., 2010; Zhang et al., 2011; Ewers et al., 2012; Johnson et al., 2012).

### Conflict of interest statement

The authors declare that the research was conducted in the absence of any commercial or financial relationships that could be construed as a potential conflict of interest.
